# The sense of agency in emerging technologies for human–computer integration: A review

**DOI:** 10.3389/fnins.2022.949138

**Published:** 2022-09-12

**Authors:** Patricia Cornelio, Patrick Haggard, Kasper Hornbaek, Orestis Georgiou, Joanna Bergström, Sriram Subramanian, Marianna Obrist

**Affiliations:** ^1^Ultraleap Ltd., Bristol, United Kingdom; ^2^Department of Computer Science, University College London, London, United Kingdom; ^3^Department of Computer Science, University of Copenhagen, Copenhagen, Denmark

**Keywords:** sense of agency (SoA), human–computer interaction (HCI), human-computer integration, body, action, outcome

## Abstract

Human–computer integration is an emerging area in which the boundary between humans and technology is blurred as users and computers work collaboratively and share agency to execute tasks. The sense of agency (SoA) is an experience that arises by a combination of a voluntary motor action and sensory evidence whether the corresponding body movements have somehow influenced the course of external events. The SoA is not only a key part of our experiences in daily life but also in our interaction with technology as it gives us the feeling of “I did that” as opposed to “the system did that,” thus supporting a feeling of being in control. This feeling becomes critical with human–computer integration, wherein emerging technology directly influences people’s body, their actions, and the resulting outcomes. In this review, we analyse and classify current integration technologies based on what we currently know about agency in the literature, and propose a distinction between body augmentation, action augmentation, and outcome augmentation. For each category, we describe agency considerations and markers of differentiation that illustrate a relationship between assistance level (low, high), agency delegation (human, technology), and integration type (fusion, symbiosis). We conclude with a reflection on the opportunities and challenges of integrating humans with computers, and finalise with an expanded definition of human–computer integration including agency aspects which we consider to be particularly relevant. The aim this review is to provide researchers and practitioners with guidelines to situate their work within the integration research agenda and consider the implications of any technologies on SoA, and thus overall user experience when designing future technology.

## Introduction

With the evolution from human–computer interaction (HCI) toward human–computer integration, the boundary between humans and computers has become blurred. Technology is increasingly becoming not only part of our daily life tasks but also of our bodies (Roggen et al., 2011). We live in a digital world in which sensors are attached, devices are worn, and intelligent algorithms assist us and influence our behaviour. Integrated technology covers devices that knowingly assist the user to achieve a goal, such as extra limbs, or mechanical actuation of the body (e.g., exoskeletons). This suggests a bodily approach that is directly associated with assisting the body’s musculature. However, integrated technology can also cover digital systems that knowingly or unknowingly influence our behaviour, such as artificial intelligence (AI) systems behind algorithmic suggestions or autocomplete predictors (Heer, 2019). This means that this integration can happen between the user and a software agent (e.g., an algorithm) or a body assistant (e.g., extra limb).

In both cases, body assistance and behaviour influence, a common aspect of human-computer integration is the mixed agency between humans and systems. The sense of agency (SoA), often referred to as the feeling of being in control, arises when a person has an intention to produce a particular *outcome*, the *body* moves by *action* of the brain’s voluntary motor system, and produces the intended *outcome* in the environment (Chambon et al., 2014). Emerging integrated technology is changing how we experience these events, as designers aim to augment how the user experiences their own *body*, the *actions* executed, and the resulting *outcomes* of these.

For example, the capabilities of the user’s *body* may be augmented using attached devices, such as when getting an extra limb (Gourmelen et al., 2019). Systems assist the user’s actions by providing a more efficient path from intention to outcome. For example, actions may be made faster (Kasahara et al., 2019), more rhythmic (Ebisu et al., 2017), or even acting on the humans behalf as in autonomous driving (Berberian et al., 2012). A system can also augment outcomes by giving the user the perception of amplified sensory features in an environment, usually in virtual reality (VR). For instance, the user may have the illusion that an object is heavier (Rietzler et al., 2018) or a room is bigger (Montano-Murillo et al., 2017).

While research has discussed the challenges around human–computer integration (Farooq and Grudin, 2016) and provided classifications of integrated systems, for instance in terms of its compatibility with humans (Mueller et al., 2020), an articulation around agency is missing in the literature. Current accounts in the literature might be confusing due to different terminology used to refer to integration such as *symbiosis*, partnership, or *fusion* (Farooq et al., 2017). We argue that by looking at integration systems through the lens of SoA, we could provide a clearer and more accurate definition of human–computer integration.

To fill this gap, we first review and classify current integration systems intro three main categories - *body augmentation*, *action augmentation*, and *outcome augmentation* (summarised in Table 1). For each augmentation category, we (1) describe how the SoA arises, (2) highlight the type of limitation the technology addresses, (3) specify where the SoA is experienced—body or eternal, and (4) illustrate a relationship of such technology with assistance level, agency, and integration type. We conclude with a reflection on the opportunities for agentic integration, some ethical challenges of integrating humans with computers, and finally, we build upon the recent views from Farooq and Grudin (2016) and Mueller et al. (2020) to expand the definition of integration including other aspects related to agency, which we consider to be particularly relevant. The aim of this review is to provide researchers and practitioners with guidelines to situate their work within the integration research agenda and consider the implications of any technologies on SoA, and thus overall user experience when designing future technology.

**TABLE 1:** Key properties of our classification.

Categories	The role of agency	Type of limitation (Scenario example)	Agency type	System sub-categories	Key examples
Body augmentation	The user’s **action** *controls* the **system** to produce an intended **outcome.** Crucially, the outcome is a body movement, or a close equivalent	The user plans an action and expects an intended outcome, but since **the user’s own body has limitations (e.g., only two arms)**, the system extends the human body so that the user experiences a match between the action and the intended outcome.	Body agency	Extra limbs Prosthetics	Arms extensions, extra fingers, tail extension, assistive finger Bionic legs, arms, eyes
Action augmentation	The **system** *assists* the user’s **action** to produce the intended **outcome**. Here also the system often goes beyond bodily limitations	The user plans an action and expects an intended outcome, but since **the user lacks the needed skills to achieve such outcome (e.g., lacking speed)**, the system helps/assists the user so that they experience a match between the action and the intended outcome.	Body agency/ External agency	Input command Motor actuation Intelligent systems	Touchpads, electrodes, epidermal electronics, brain interfaces Electric stimulation, exoskeletons Autocompletion predictors, autonomous driving, autoplay features
Outcome augmentation	The **system** *modulates* the beliefs of the environment where the **outcome** occurs to match the **user**’s intention	The user plans an action and expects an intended outcome, but since **the environment cannot offer such outcome (e.g., due to constraints in the physical space)** the system detects such limitations and adjusts the experienced environment in a way that the user experiences a match between the action and the intended outcome.	External agency	Illusions in VR Illusions in real environments	Haptic retargeting, redirected walking, infinite walking, translational gains, tracking offsets Crossmodal associations, multisensory information that influences perception

## The sense of agency

The SoA refers to the experience of being the initiator of one’s own voluntary actions and through them influencing the external world (Beck et al., 2017). Georgieff and Jeannerod (1998) defined this phenomenon as a “who” system that permits the identification of the agent of an action and thus differentiates the self from external agents.

Unlike views of agency related to *beliefs* or felt capacity to act (e.g., the sense of self-efficacy described by Bandura, 1982), the SoA rather refers to the *experience* that is associated with actual goal-directed motor acts, that is, the body moving under intentional control, to achieve the goal state (Haggard, 2017). To illustrate this difference, a person may have agency beliefs over some aspect of their lives (“I could lift that weight”) but might not actually do anything to bring this about. In contrast, if the person moves their body and succeeds in lifting the weight, they are assumed to have SoA with respect to the corresponding displacement of the weight. Agency beliefs may be background, non-event-related and counterfactual, whereas SoA is strictly factual and event-related.

**FIGURE 1:**
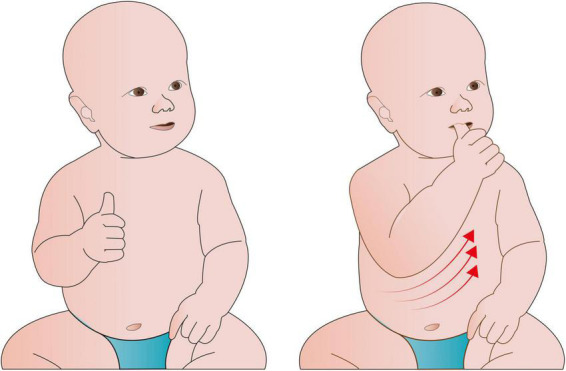
Humans experience a SoA from an early age. Infants gradually develop control over their own body in simple actions. The fact that some action patterns, such as thumb sucking, are increasingly repeated suggests that the infant may experience a link between their control of the action and the rewarding sensation that the action produces.

The SoA reflects the experience that links intentions to their external outcomes. The match between the intended and actual result of an action produces a feeling of being in control (Synofzik et al., 2013). The brain mechanisms that produce this experience are quite efficient and familiar, so that our SoA is experienced naturally and like a continuous mental background during everyday motor movements. Indeed, we acquire a SoA over our own bodies from an early age. Studies show evidence for the early presence of a SoA in infants as young as 2 months of age for some actions such as smiling and thumb sucking (Rochat and Striano, 2000; Figure 1).

While the SoA begins when we are infants with the sensorimotor experience of controlling our own body as shown in Figure 1, humans are able to transfer a SoA from one’s own limb to objects or events, external to the body (Caspar et al., 2015). For example, when the expected outcome occurs in the external world, such as happens when we drive a car and perceive the car turning after moving the steering wheel (e.g., “I control this”). In other words, we use our bodies to control the external world, the SoA over our limbs, transfers to SoA over the objects our limbs interact with. Presumably, this just reflects associative plasticity in the brain (Iriki et al., 2001).

**FIGURE 2:**
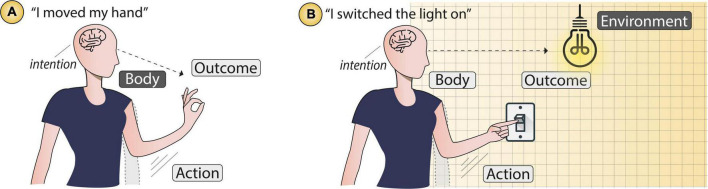
Elements that compose the SoA. An intention to produce an outcome followed by the body moving to perform the action that produces such outcome. **(A)** *Body agency*: the outcome occurs in the body itself (mainly movements). **(B)** *External agency*: the outcome occurs outside the body (in the environment).

Therefore, the SoA contains two layers, *body agency* and *external* agency (Wen, 2019). The first is illustrated in Figure 2A, and refers to the experience of controlling one’s own body, and receiving the bodily feedback that results from the movement one had commanded (e.g., moving my hand). The second is illustrated in Figure 2B, and refers to the experience of controlling external events and receiving the appropriate external feedback from the environment (e.g., switching the light on). We mark this difference as we use these two layers in our classification in later sections.

In both cases, to experience a SoA three conditions need to occur, (1) one intends to produce an *outcome* through one’s own *action*, (2) one voluntarily commands the corresponding *body* movement, and (3) the intended *outcome*, either in the body itself, or in the external world, occurs. These conditions are present during our everyday life as we constantly perform goal-directed motor actions and we observe the consequences of those actions (Hommel, 2017). In such cases, we readily recognise that our voluntary actions cause external effects (e.g., the illumination of the room in Figure 2B). The SoA is the experiential aspect of this fact. This action-effect causality is particularly crucial in our interactions with the technologies that figure in HCI.

**FIGURE 3:**
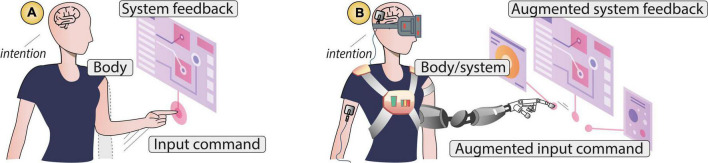
**(A)** Human–computer interaction—a stimulus–response interplay between humans and technology, **(B)** human–computer integration—a symbiosis/fusion in which humans and technology share agency augmenting the capabilities of body, action, and outcome.

Human–computer interaction is defined as a stimulus–response interplay between humans and technology (Farooq and Grudin, 2016). Actions are represented by user input commands, and outcomes are represented by system feedback. Input modalities thus serve to translate user’s intentions into state changes within the system, while system feedback informs the user about the system’s current state (see Figure 3A). Here, the SoA is crucial to support a feeling of being in control. For instance, when we manipulate a user interface (e.g., on a computer or smartphone), we expect the system to respond to our input commands as we want to feel we are in charge of the interaction. If this stimulus–response interplay elicits a SoA, then the user will have a feeling of “I am controlling this.”

Due to the ubiquity of our interaction with systems for work or leisure purposes, we usually do not think about our SoA during the interaction with technology, and it may go unnoticed (Moore, 2016). However, a clear example of the importance of our SoA in HCI is when this experience is disrupted. When there is a mismatch between what the system is expected to do and the actual sensory feedback from the system, the user experiences a sudden interruption in the feeling of control. This can negatively affect acceptability (Berberian, 2019) and usability (Winkler et al., 2020). For example, poor game controllers may cause frustration (Miller and Mandryk, 2016). Moreover, if a system does not support a SoA, then the user might feel discouraged from using it (Limerick et al., 2015) and lose self-attribution of their actions’ outcomes.

Loss of SoA during interactions with technology is commonplace (try buying a train ticket when you first arrive in a new country). However, technology should, in principle, be able to enhance the SoA, rather than frustrate or reduce it. For this reason, SoA is gaining increasing attention from the field of HCI. Designing interactions that increase the user’s SoA will provide the feeling of “I did that” as opposed to “the system did that,” thus supporting a feeling of being in control.

With the increasing ubiquity of technology, advances in bio-sensing, intelligent systems and the digitalisation of the human senses (Velasco and Obrist, 2020), there is an evolution from HCI, toward human–computer integration, in which the boundary between humans and computers becomes blurred. While this evolution represents a great advancement for assisting humans in daily tasks, work, and leisure, the impact of such integration on the SoA has been less studied. These situations are sometimes referred to as *shared agency* in which both systems and humans have control over the technology (Wen et al., 2019). However, this term can be deceptive. If the user is to feel a strong and convincing SoA, we argue that events must follow from the user’s intentions, not from those of any other agent. Thus, true shared agency would seem to require the system to understand the user’s intentions, and align with them, facilitating them rather than frustrating them. Next, we describe the challenges of human–computer integration.

## Human–computer integration and the role of agency

Unlike HCI, human–computer integration refers to a partnership in which humans and systems act with autonomy. A characteristic of such integration is the designers’ goal to augment the capabilities of the user’s own *body*, the *actions* executed, and the *outcomes* resulting from those actions. For example, systems can augment the user’s body by adding an extra limb that makes actions faster, also resulting in amplified sensory outcomes (see Figure 3B). We define augmentation technology as integration systems that aim to enhance the path from intention to outcome, addressing current limitations from the user’s body or the environment where the outcomes occur.

A major issue in human–computer integration is the mixed agency between humans and technology. Today, multisensory technology is becoming more connected to our body, emotions and actions, since sensors can be worn and allow mobile interactions in daily activities (Zhu et al., 2014). Feedback from systems is mediated by the user’s biological responses and emotional states (Amores and Maes, 2017). Virtual environments enable the embodiment of virtual avatars, thus creating the feeling of body ownership, with realistic environments no longer limited to audio-visual experiences but also including touch (Sand Rakkolainen et al., 2015), smell (Ranasinghe et al., 2018), and taste experiences (Narumi et al., 2011). There is also increasing efforts to integrate humans and robots by designing robotic systems inspired by human biological systems (Pfeifer et al., 2012). For example, designing robots with soft materials and body morphology (Pfeifer et al., 2014). Indeed, Pfeifer and Gómez (2009) have illustrated the concept of morphological computation which is about connecting body, brain and environment.

There are different views to describe human–computer integration in the literature. For example, Mueller et al. (2020) describe integration as a sensory “*fusion*” that involves biosensing in which actuators are attached/implanted to the user’s body and communicate directly to human senses rather than through symbolic representations. This definition suggests that technology becomes physically part of the user’s body. In this integration type, agency is shared as the user’s actions are physically assisted by the system to obtain a more efficient path from intention to outcome. Some examples of *fusion* described by Mueller et al. (2020) are extra limbs. For instance, a robotic prosthesis which circuits are connected directly to the human nerves and communicate with the user’s motor system allowing the user to freely control the robotic prosthesis.

Moreover, Farooq and Grudin (2016) describe integration as a “*symbiosis*,” in which there is not necessarily a physical attachment of devices to the user’s body, but agency is shared between humans and digital systems as they assist or work on the humans’ behalf even when the human is not attending them. In this integration type, although there is not physical body actuation, agency is shared as the user’s actions are influenced by the system to obtain a more efficient path from intention to outcome. Some examples of *symbiosis* described by Farooq and Grudin (2016) are AI systems such as autonomous driving or intelligent rescheduling of meetings. In both integration types, the system always prompts the user to make an action, involving thus a joint action. We use the terms “*fusion*” and “*symbiosis*” to differentiate our classification in later sections.

An increased integration leads to the challenge of a shared agency between humans and digital systems (Cross and Ramsey, 2020). Current technology often posits the user in environments that are not fully real (e.g., virtual or augmented) and where the user’s actions are sometimes influenced (e.g., autocompletion predictors) or even automated (e.g., autonomous driving) and therefore the feeling of being in control can be challenged. For example, it has been shown that autoplay features and recommendations can reduce the SoA (Lukoff et al., 2021). Notwithstanding, emerging research is committed to improve the SoA for human–computer integration technology. For example, by designing motor actuation without diminishing the SoA (Kasahara et al., 2019) or exploring appropriate levels of automation (Berberian et al., 2012). Despite such efforts, it has been suggested that “the cognitive coupling between human and machine remains difficult to achieve” (Berberian, 2019).

While prior work has discussed the challenges around human–computer integration, an articulation of the key challenges around agency is missing in the literature. To help practitioners interested in agency implications for human–computer integration, this paper reviews emerging integration systems and classifies them intro three main categories—*body augmentation*, *action augmentation*, and *outcome augmentation* (summarised in Table 1). In each category we discuss how agency is shared between the user and the system and provide discussion that we hope can serve as guidelines for agency implication when designing future integration technology.

## Our approach

We analyse integration systems that share agency with the user putting special attention on the psychology aspects of the technology, rather than in the engineering advances. However, our review already gathers a large number of innovations on integration, that readers can use for being up to date. The works reviewed here do not necessarily measure or show results on SoA. Indeed, most of the technology discussed in this paper does not consider implications for agency in its design or evaluation. This is part of our motivation arising from the lack of focus on agency in the field of HCI and recently in human–computer integration.

To that end, based on what we know about agency in the literature (e.g., how it arises) and the characteristics of the technology (e.g., sharing control with the user), and motivated by the key elements of SoA (see Figure 2), we have created these three categories as summarised in Table 1 and expand them in terms of agency type, limitations, and scenarios. To ensure we consider only technologies involving SoA, we included only systems that meet the following two criteria:

• *Motor target*: The device or technique needs to involve a movement from the user and not simply beliefs about potential actions. That is, the action might start by a simple thought or intention but needs to wind up in an actual motor movement. Therefore, we do not consider passive assistive technology such as music recommendations.
• *Intentional actions*: Only systems involving voluntary movements are considered. Even if movements are assisted, they should at least involve intentions or pre-planning from the user. Therefore, technology involving completely passive assistance that actuates/assist the user without the user’s intention are not considered (e.g., an implanted heart pacemaker).

We created our three categories based on three main elements that compose the SoA and that are key in human–computer integration (i.e., *body*, *action*, and *outcome*). We refer to *body* as the physical structure that acts via somatosensory inputs (Serino and Haggard, 2010), *action* as the process to achieve an aim including motor preparation, specification of motor commands and sensory feedback from actual body movement (Haggard et al., 2002), and *outcome* as the result of the action. We refer to system as the technology, device, or technique (within our inclusion criteria) that facilitates the match between the intention and the outcome. Finally, we refer to environment as the physical space where the outcomes occur (as opposed to internal outcomes occurring inside one’s own body).

Below we describe markers of distinction of the different categories (rather than in their similarities or overlaps) in order to identify aspects that delimit augmentation type (*body*, *action*, and *outcome*). Based on the literature review, we found three main distinctions:

1. *The role of agency during the interaction*: This refers to how SoA arises during the interaction and the type of control that the user has over the system and vice versa, to produce a match between the user’s intention and the intended outcome (see column 2 in Table 1). For example, in the *body augmentation* category, the user has a higher level of influence over the system, as the user has full control to produce the outcome (e.g., controlling the movement of an extra limb). In the *action augmentation* category, the system has more influence over the user, as it assists them to achieve a desired outcome which might not be achieved without the system’s assistance (e.g., accelerate the user speed to catch an object). In the *outcome augmentation* category, the system has more influence over the outcome than the user has, as it modulates the experienced result of an action without the user realising (e.g., a VR system that creates the illusion that the environment is larger).
2. *The limitations that the technology addresses*: The integrated technology comes into play depending on the origin of the limitation (see column 3 in Table 1). For example, in the *body augmentation* category, technology helps in the limitations of the user’s own *body* (e.g., number of limbs). In the *action augmentation* category, technology addresses the limitations of the user’s *skills* (e.g., the user dexterity to perform actions). Finally, in the *outcome augmentation* category, the technology addresses the limitations of the experienced environment where the outcomes occur (e.g., the physical space to interact with). In other words, technology assist when the user’s goal is basically not achievable in pure form.
3. *Where the agency is experienced*: This refers to whether the user experiences a *body agency* or an *external agency* (see column 4 in Table 1). For example, the *body augmentation* category includes extra devices that resemble the user’s own body, and therefore the user mostly experiences a SoA over *body* movements. The *action augmentation* category includes systems that involve assisted motor actions that produce outcomes in the environment, and therefore the user can experience a SoA of both their own *body* movements and *external* events. Finally, the *outcome augmentation* category includes systems that modify the experienced external environment and therefore the user mostly experiences a SoA over *external* events rather than over body movements.

### Body augmentation (motor attached technology)

The SoA reflects an experience of control over one’s own body. However, feeling that “my body moved” is not sufficient. We need to experience the voluntary experience of “I made my body move” to have a SoA (Haggard, 2017). The emerging area of human–computer integration is changing the way people control technology with their own body. For instance, wearable devices and prosthetics can extend the user’s body, not only by resembling the human shape but also by replicating human movements, giving users a high level of control. This creates a shared experience between the user and the system that we call *body augmentation*.

**FIGURE 4:**
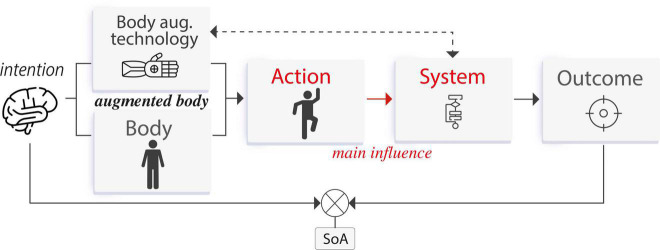
Agency process for *body augmentation* technology. The user’s action controls the system to produce an intended outcome. Crucially, the outcome is a body movement.

*Body augmentation* technology aims to amplify the physical attributes of the user’s body to achieve a desired goal, in which outcomes involve the body itself (*body agency*). That is, the outcome is a body movement exclusively. These devices are perceived as part of the user’s own body, and whose movements can be directly controlled by the user. During the interaction with this technology, the SoA arises by a process in which the user’s actions *control* the system to produce an intended outcome by an initial motor movement (or an intention to move), which is then processed by the system to produce the expected outcome. Such outcome is observed by the user and compared with the intention, if there is a match, then a SoA occurs. The main interaction is given by the influence that the user has over the system (see Figure 4).

*Body augmentation* technology addresses limitations of the user’s own body by augmenting its attributes (e.g., number of limbs). For instance, the user plans an action and expects an intended outcome (e.g., touch three objects at the same time), but since the user’s own body has limitations (e.g., only two arms), this technology extends the human body (e.g., giving an extra arm) so that the user experiences a match between the action and the intended outcome. Although the user knows that system might not be part of their own body (e.g., “this is a robotic arm”), it can be controlled by the user in visuomotor synchronisation enabling a feeling of body ownership, and therefore the user experiences a SoA. Behavioural accounts have demonstrated that body ownership can be extended to external objects (Tsakiris and Haggard, 2005) such is the case of tool use (Martel et al., 2016). This effect allows people to experience a SoA even when a body extension is external and does not necessarily resemble a human shape.

**FIGURE 5:**
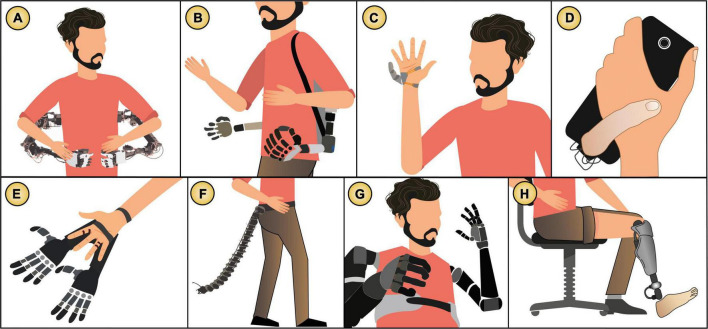
Examples of *body augmentation* technology: **(A,B)** Arms attached to the user’s body (Sasaki et al., 2017; Gourmelen et al., 2019), **(C)** bionic second thumb (Kieliba et al., 2021), **(D)** extra finger assisting phone interactions (Teyssier et al., 2018), **(E)** double bionic hand (Youbionic, 2017), **(F)** tail extension (Svanaes and Solheim, 2016; Nabeshima et al., 2019), **(G)** prosthetic bionic arms (Canepari, 2015), and **(H)** leg (Demarco, 2015). Images based on the original publications.

**FIGURE 6:**
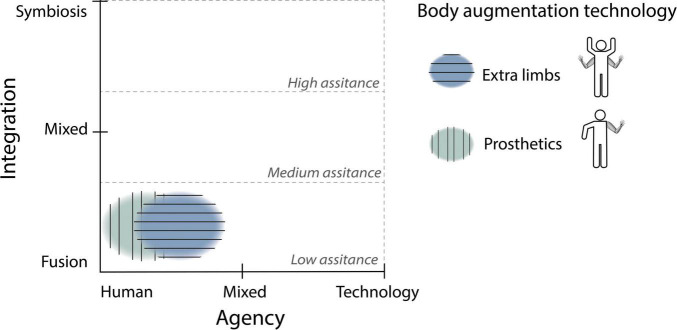
Map of the three types of *body augmentation* technology that we identified (extra limbs and prosthetics) and their relationship with assistance level, agency, and integration.

Crucially, *body augmentation* devices produce *body agency*, replicating the biological properties of the human body (Laffranchi et al., 2020), such as movement, kinaesthesia or touch (see Figure 5). For example, a second thumb in my hand, whose movements I can control just like the rest of my fingers (Kieliba et al., 2021), or a prosthesis replacing an amputated arm that gives me the perception of touch and pain (Osborn et al., 2018). This type of integration has been named *fusion* (Mueller et al., 2020) as there is an embodied mediation, in which technology is attached, implanted or wearable. Particularly, *body augmentation* technology delegates the user full agency as these devices offer low assistance to the user. Figure 6 shows a map of the two types of *body augmentation* technology that we identified (extra limbs and prosthetics) and their relationship with assistance level (low-high), agency delegation (human-technology) and integration type (fusion-symbiosis). In the next section, we provide more examples of integration technology that add (extra limbs) and replace (prosthesis) body parts to the user.

#### Extra limbs

People can experience body ownership and agency over virtual extra limbs (Hoyet et al., 2016; Chen et al., 2018). For example, studies suggest that for demanding tasks, “three-handed manipulation is preferred to two-handed manipulation” (Abdi et al., 2016). Imagine a surgeon performing a crucial surgical intervention with three hands when help is not available. Advances in computer science, robotics and artificial intelligence are making possible the vision of humans with extra limbs beyond VR. For example, Gourmelen et al. (2019) proposed collaborative limbs controlled by joysticks expanding the interaction to four arms, which are able to learn and replicate the user movements (see Figure 5A). Similarly, Sasaki et al. (2017) designed two robotic extra arms with voluntary control using legs motion mapping (see Figure 5B).

Supernumerary robotic fingers are also found in the literature. For example sixth finger approaches (Prattichizzo et al., 2014; Wu and Asada, 2014; Hussain et al., 2016), that use control algorithms enabling the extra and human fingers to share movements. More recently, Kieliba et al. (2021) developed an extra robotic thumb that is controlled by pressure exerted with the big toes, designed to extend the natural repertoire of hand movements (see Figure 5C). Artistic projects have been also proposed, which involve body extensions such as mechanical ears and a tail extension controlled by body movements (Svanaes and Solheim, 2016) as shown in Figure 5F.

A SoA can also be experienced for extra limbs that are not necessarily attached to the body but still resemble some humanistic features extending the body schema (Ataria, 2015). Research on tool use shows that the body can be extended to external objects such as a drumstick acting as a finger (Kilteni and Ehrsson, 2017) or computer-based tools (mouse and touchpad) acting as hand input modalities (Bergström et al., 2019). For example, the device by Teyssier et al. (2018) consists of a finger that although is attached to a mobile phone and not to the user’s body, it acts as an extra user’s thumb in phone interactions (see Figure 5D). Similarly, Penaloza and Nishio (2018) proposed a non-invasive BMI to control a human-like robotic arm attached to a chair.

We recall that *body augmentation* technology produces *body agency* (being outcomes mainly movements). Although detached devices might not be seen as part of the user’s own body (e.g., a finger on a phone or an arm on a chair), those devices are able to extend the body schema (Hoffmann et al., 2010). That is, they resemble the user’s body features and operations acting as part of the user’s own body. Additionally, the device movements are a consequence of the user’s own movements (or intentions), in which agency is fully delegated to the user (see Figure 6). Those are examples of systems in which the user transfers agency to objects external to the body (Caspar et al., 2015), but still are considered *body augmentation* technology. Thanks to the availability 3D-printing technology, different *body augmentation* devices are commercially available for purposes of extra limbs (Youbionic, 2017) and prosthesis (Liarokapis et al., 2014).

**Scenario example** | The user has a motor disability that constrains them from moving their arms and legs and therefore cannot control a conventional electric wheelchair (e.g., joystick-based) see Figure 9B, but they can perform some subtle facial expressions. To assist the user, the system detects small muscle movements from the user’s face (recorded by sensors located on the user’s cheeks) and translate them into patterns to control the wheelchair. This input produces an expected outcome (e.g., the extra arm moving to the left) that gives the user an experience of controlling the robotic arm.

**FIGURE 7:**
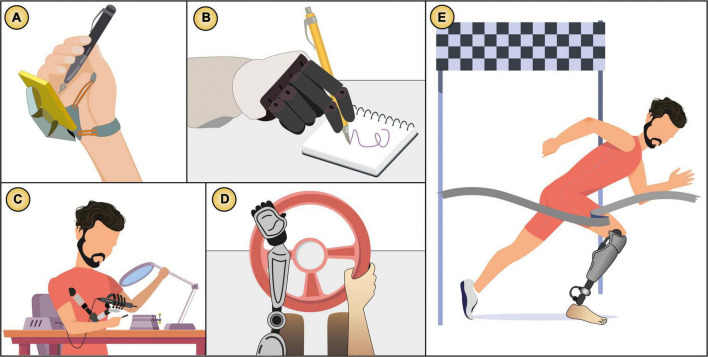
Examples of body augmentation technology that becomes action augmentation technology. Extra limbs and prosthetic technology that not only provide motor control but also help the user to perform complex tasks and achieve goals in the external world. **(A)** Royal College of Arts (2021), **(B)** Open Bionics (2021), **(C)** Sasaki et al. (2017), **(D)** Arm Dynamics (2020), **(E)** Prosthesis used in the Paralympics. Images based on the original publications.

**FIGURE 8:**
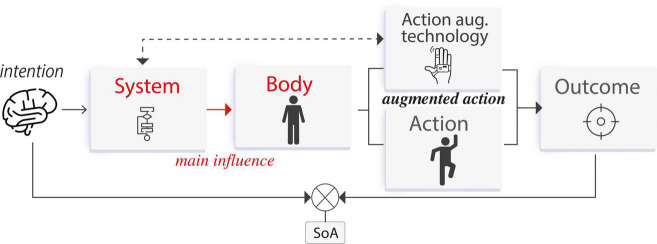
Agency process for *action augmentation* technology. The system assists the user’s action to produce the intended outcome. Here also the system often goes beyond bodily limitations.

**FIGURE 9:**
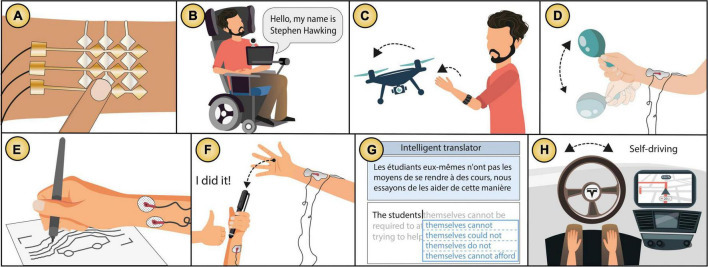
Examples of *action augmentation* technology: **(A)** Skin electronics that make the user’s skin an input modality (Kao et al., 2016), **(B)** a text entry system controlled by facial expressions (Hemsley, 2018), **(C)** a drone controlled through EMG sensors (Hockett, 2017), **(D–F)** electric muscle stimulation that assists in **(D)** rhythmic tasks (Ebisu et al., 2017), **(E)** plotting tasks (Lopes et al., 2016), **(F)** reaction time acceleration (Kasahara et al., 2019), **(G)** an autocompletion predictor to translate text (Heer, 2019), and **(H)** a Tesla vehicle in autopilot mode (Tesla, 2022). Images based on the original publications.

#### Prosthesis

Amputees can experience a SoA over missing limbs, and this experience can be transferred to a prosthetic limb even when this does not necessarily look human (e.g., robot-like advanced hand prosthesis, Rosén et al., 2009). For instance, prior evidence shows that patients with poor motor movement report a feeling of agency for actions using neuro-controlled prostheses (Hochberg et al., 2006). To successfully use an advanced prosthetic limb the user needs both effective motor control and sensory feedback. Today, there are techniques that not only control motorised joint movements but also provide kinaesthetic perception of dexterous robotic hands via a neural-machine interfaces (Marasco et al., 2018).

*Body augmentation* technology aims to control prosthetics in concert with the user’s intentions (Tucker et al., 2015). Some examples are bionic prostheses able to recognise the user’s intended movement, translate the intended movement into an appropriate pattern of limb movement and execute the desired motions with closed-loop control. These devices can be controlled by EMG activity (Furui et al., 2019), intramuscular sensors and nerve transfers (Hargrove et al., 2013; Salminger et al., 2019), as well as neurostimulation (Valle et al., 2021). Some of these devices are even able to restore tactile sensations (Osborn et al., 2018; Zollo et al., 2019). Some devices are able to provide multisensory continuous multisensory feedback required for a limb to be experienced as one’s own (Rognini et al., 2019).

In summary, we argue that *body augmentation* technology amplifies the processes of the body itself, such as the user’s movements or the sense of touch (*body agency*), by means of attached devices or by extending the body to external devices, but not necessarily augments the user’s actions that produce external events in the outside world (*external agency*). When a device assists the user’s actions, thus enhancing limited skills (e.g., improving speed or dexterity), we call it *action augmentation* (explained in next section).

For example, a robotic arm can extend the user body, which movements can be fully controlled by the user “I voluntarily made it move” and produce a SoA, but if the same robotic arm assists the user to make more complex tasks, in which the outcome occurs outside the body (e.g., drawing an artwork, or soldering more precisely, as shown in Figures 7B,C), then that system becomes an *action augmentation* technology. In another example, a bionic limb can extend the user’s own body by replacing an amputated leg that can be controlled by EMG to walk, but if the same device helps the user to drive a car or win a golden medal in the Olympics (see Figures 7D,E), then that system becomes an *action augmentation* technology as well.

This means that one single device can be considered at the same time under both types of augmentation *body* and *action*. However, we define the limits of the *body augmentation* to the outcomes being internal body processes (*body agency*), while *action augmentation* enables outcomes outside the body as well (*external agency*). Figure 7 shows examples of devices that share *body* and *action* augmentation.

### Action augmentation (systems assisting human action)

The SoA arises for voluntary actions that cause an outcome in the environment. An experience of action can include intentions (a conscious thought before action, Haggard, 2005), decisions (choosing to make one particular action rather than another, Schultze-Kraft et al., 2016) and motor movements (the body actually moving, Haggard, 2017). For involuntary actions however (e.g., reflexes evoked by brain stimulation), the SoA does not occur (Moore et al., 2009). In the emerging area of human–computer integration, technology designers and researchers aim to augment the capabilities of action, reflecting a shared agency between the user and the system, we call this *action augmentation*.

*Action augmentation* refers to technology that assists the user in executing motor actions to achieve a desired goal, in which an outcome can produce *body agency* or *external agency*, unlike *body augmentation* technology where outcomes produce exclusively *body agency*. This includes systems that assist the user not only by improving the result of a voluntary input command or by directly actuating the body muscles, but also by influencing their decisions. During the interaction with this technology, the SoA arises by a process in which the system knows or predicts an intended outcome and then *assists* the user’s actions to produce such outcome without diminishing the experience of agency. The outcome is observed by the user and compared with the intention, if there is a match, then a SoA occurs. The main interaction is given by the influence that the system has over the user (see Figure 8). Examples of *action augmentation* technology are attached actuators that move the user’s body or even algorithmic suggestions that influence what the user intends to do (see Figure 9).

While research suggests that assistance can negatively affect the SoA (Berberian et al., 2012; Le Goff et al., 2018; Berberian, 2019), studies have shown that giving assistance improves user performance, which in turn produces a positive effect on agency (Wen et al., 2015; Inoue et al., 2017). Therefore, *action augmentation* technology aims to increase the perceived user’s performance by giving assistance but without diminishing the SoA.

In contrast to *body augmentation*, in which technology addresses limitations of the body itself, a*ction augmentation* technology addresses limitations of the user’s skills by augmenting its physical capabilities (e.g., dexterity, communication). That is, the user plans an action and expects an intended outcome (e.g., play the drums), but since the user lacks the needed skills to achieve such outcome (e.g., lacking rhythm), the system assists the user so that they experience a match between the action and the intended outcome. Although the user might clearly realise that is being assisted, the interaction always involves (1) a previous intention to act (Chambon and Haggard, 2013), and (2) a feeling of good performance/accomplishment (Wen et al., 2015) and therefore they experience a SoA.

While *action augmentation* technology can be seen as systems that might change the course of the user’s action, this technology only changes the action to match the intention, in the absence of the necessary skills. For example, a person might want to walk but do not be able to due to a motor disability, then the technology (e.g., an intelligent exoskeleton) augments subtle motor movements (that without the technology assistance are not enough to meet the intention) to achieve the ultimate goal—to walk.

**FIGURE 10:**
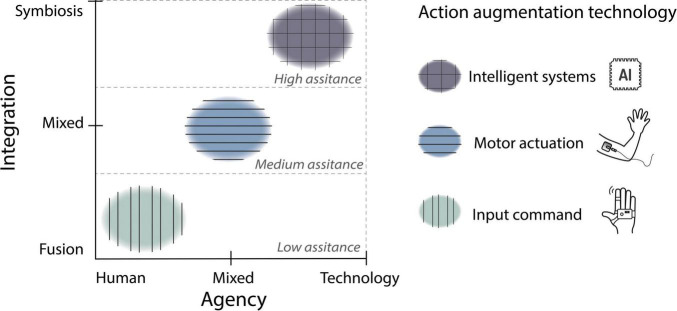
Map of the three types of *action augmentation* technology that we identified (input command, motor actuation and intelligent systems) and their relationship with assistance level, agency, and integration.

We identified three types of *action augmentation* technology, (1) *Input command*: integrated systems that augment an entered motor command (e.g., touch, gestures, voice, EMG activity), in which the user has full agency (see Figures 9A–C). (2) *Motor actuation*: integrated systems that actuate the user’s muscles (e.g., by means of electric stimulation), in which agency is shared between the user and the system (see Figures 9D–F). (3) *Intelligent systems*: integrated systems that have humanistic intelligence (Mann, 1998) and influence the user behaviour or act on behalf of the user (see Figures 9G,H). See Figure 10 for a map of the three types of *action augmentation* technology that we identified and their relationship with assistance level (low-high), agency delegation (human-technology) and integration type (fusion-symbiosis).

#### Input command

On-skin interaction, also called *Skinput* (Harrison et al., 2010) or skin computing (Steimle, 2022), is a technique that uses body landmarks (Steimle et al., 2017) or wearable bio-acoustic sensors on the user’s body, allowing the skin to be used as a finger input surface. Indeed, it has been suggested that on-skin input produces a higher SoA compared with traditional button-press (Coyle et al., 2012) and touchpad (Bergstrom-Lehtovirta et al., 2018a) inputs.

This approach has led to epidermal electronics that integrate the user with a variety of sensors (e.g., temperature and strain sensors) on the skin in form of lightweight tattoos (Weigel et al., 2015, 2017; Lo et al., 2016). This technology has been claimed to be easy to prototype with skin-friendly materials (Kao et al., 2016), which are soft and stretchable—like the human skin (Ma, 2011; Wang et al., 2018; Nittala et al., 2019; see Figure 9A). Different studies have been conducted to understand the mapping between on-skin input and outcomes in the external environment such as displays (Bergstrom-Lehtovirta et al., 2018b), and gaming (Zhang et al., 2016), giving the user great levels of control over external events triggered by their own skin.

Gestural interaction is also a common input modality achieved via sensing the user’s body input, to control external devices or events (see Figure 9C), such as drones (La Delfa et al., 2020), video games (Tang et al., 2011), in-vehicle controls (Young et al., 2020), multimedia (Vo et al., 2014) among others. Some of these wearable devices can even provide haptic feedback on the skin as outcome confirmation (Prattichizzo et al., 2013; Ramachandran et al., 2021). Indeed, gestural input has been shown to provide a SoA even though it does not have the typical characteristics of physical interaction (e.g., pressing a button) (Cornelio et al., 2017).

Since this technology aims to augment the user’s actions (addressing potential limitations), different applications have been directed to disabled people. For example, those motor or communication disabilities can benefit from a small input command (e.g., a subtle face gesture or a tongue movement, detected by attached sensors) that is processed by the system to produce a more complex or amplified outcome, such as controlling the direction of a wheelchair (Jia et al., 2007; Kim et al., 2013) or typing sentences (Taylor, 2009), see Figure 9B. Wearable sensors can also help in gait assistance for Parkinson’s disease patients (Mazilu et al., 2014).

**Scenario example** | The user has a motor disability that constrains them from moving their arms and legs and therefore cannot control a conventional electric wheelchair (e.g., joystick-based) see Figure 9B, but they can perform some subtle facial expressions. To assist the user, the system detects small muscle movements from the user’s face (recorded by sensors located on the user’s cheeks) and translate them into patterns to control the wheelchair. This input produces an expected outcome (e.g., the wheelchair decreasing its speed) and then the user experiences a SoA.

One particular characteristic of the aforementioned technology is that although the input commands might be simple (as they will be augmented), the user the suer is delegated full agency over the actions executed, which means that there is low or null assistance from the system. Moreover, this type of integration is considered as a *fusion* (Mueller et al., 2020) as there is an embodied mediation, in which technology is attached or wearable (see Figure 10).

#### Motor actuation

Unlike the integrated technology described in the previous section (in which the user in delegated full agency over the input command), motor actuation technology assists the user collaboratively, usually by means of electrical muscle stimulation—EMS (Knibbe et al., 2018). This type of integration is considered a mix of *fusion* and *symbiosis* (Mueller et al., 2020) as there is an embodied mediation in which devices are attached and agency is shared between the user and the system by assisting or working on the humans’ behalf (as shown in Figure 10).

That is, the user receives assistance from the system, by actuating their muscles, to execute actions, but the user always has an intention and acts in conjunction with the system (mixed agency). For example, Kasahara et al. (2019) explored the extent to which EMS (applied through attached electrodes to the user’s wrist) can accelerate reaction time of an action (tapping a target on a tablet) without diminishing explicit judgements of agency. They identified a particular time window, in which the action can be speeded up, making the user’s reaction time faster than usual, while still preserving a SoA (see Figure 9F).

Similarly, the muscle plotter by Lopes et al. (2016), uses EMS (applied through attached electrodes to the user’s forearm) to assist in pen-on-paper interaction by steering the user’s wrist, for drawing charts and widgets with greater accuracy (see Figure 9E). Moreover, Colley et al. (2018) explored co-creating visual art using electrical stimulation in the user’s arm. Ebisu et al. (2017) explored “stimulated percussions” to assist musical performers to produce rhythms correctly via EMS. The user’s arms and legs are equipped with electrodes that actuate the user’s body to reproduce the correct movement when they play instruments (see Figure 9D). In another example, Andres et al. (2018) explored “integrated exertion” to assist eBike riders in speed control allowing users to control the eBike’s engine acceleration when leaning forward and slowing down when standing up. Other *action augmentation* technologies involve higher assistance from the system. For example, the Ping Body is a body expression artwork by Stelarc (1996), that consists of various electrodes attached to a performer’s body to actuate their muscle movements via EMS in a way that minimal agency remains with the human. Human-robot interaction is another example of assistive technology in which agency is shared between the user and systems (Beckerle et al., 2017), for which control sharing methodologies have been proposed to explore how control should be shared among them (Music and Hirche, 2017). This interaction has been suggested to produce a positive impact on feedback loops and embodiment (Beckerle et al., 2019).

In summary, *action augmentation* technology using motor actuation aims to augment the user’s actions increasing thus the perceived user’s performance by giving assistance but without diminishing the SoA.

**Scenario example:** The user is asked to catch a pen (see Figure 9F), but they are too slow to catch it on time. To assist the user’s action (close their hand in the right moment), an electrode attached to the user’s forearm produces a small electric shock which causes their hand to close at the exact moment that the object is in front of their hand (i.e., speeding up their reaction time). This system assistance is in turn accompanied by the user’s intention to close her hand, and therefore, they believe they have made the action and their SoA is not lost.

#### Intelligent systems

Unlike motor actuation, in which the collaboration between humans and systems is physical (e.g., actuating the user’s muscles or exertion) and devices and sensors are attached (electrodes, wearables, tattoos), intelligent systems share agency on a cognitive level. That is, the system can influence our decisions, so that the action is augmented but without diminishing the SoA. This type of integration has been named *symbiosis* (Mueller et al., 2020).

Farooq and Grudin (2016) describe this *symbiosis* for digital systems that “continuously work on the human’s behalf, even when the human is not attending them.” This refers to Mann’s vision of *humanistic intelligence* (Mann, 2001), where there is a continuous feedback loop between a human and a digital system, each augmenting the other. That is, agency is shared between technology and humans acting in concert by collaborating in different tasks (Goel and Rugaber, 2015; Bretan and Weinberg, 2017; Oh et al., 2018).

Some examples of *symbiosis* described by Farooq and Grudin (2016) are Artificial Intelligent (AI) systems that execute actions on behalf of the user. This goes beyond simple reminders (a calendar agent that reminds you that today is your friend’s birthday) but they actually change or influence the course of the user’s actions. For instance, an intelligent alarm that wakes you up 15 min earlier the time you had set, because it detects that today’s bad weather will require you extra time to make sure you make it for your meeting at 8:30am, or an intelligent agent in a tablet that requires a child to finish an academic task before allowing them to watch their favourite video cartoons.

It is important to highlight however, that although autonomous systems might change the user’s actions or decisions, the ultimate goal or intention remains the same. For example, AI predictors used in translation and browsing tools, provide the user with text recommendations that suggest refinements of the user’s action. These suggestions might differ from the user original action, however since text recommendations can be quite precise (e.g., using a large database), the user can easily agree and accept such suggestions and still attribute the outcome to themselves resulting in a SoA being experienced (e.g., “I translated this text”).

A common criticism of intelligent systems is that they tend to reduce the SoA. That is, automation tends to disrupt operators from action outcomes (Le Goff et al., 2018), and clickbait and autoplay features can “exploit psychological vulnerabilities to maximise watch time” (Lukoff et al., 2021) in the designers’ pursuit to achieve attention economy (Davenport and Beck, 2001). Since these features prompt the user to act in a way they might not do without them, some studies suggest that social media reduce the SoA (Baumer et al., 2018). There are also fully automated systems, that serve as a replacement for human labour in which agency is completely delegated to technology.

Therefore, *action augmentation* technology using intelligent systems needs to be carefully designed to preserve a SoA and avoid giving the user a feeling that the system is acting by its own. Different methods have been proposed to preserve a SoA for intelligent systems. For instance, letting the user know the systems’ intentions (Le Goff et al., 2018), giving the user the ultimate decision on what to do (Heer, 2019) or regulating the level of assistance (Berberian et al., 2012). Some examples of intelligent systems that preserve the user’s SoA are cars, machinery and aircrafts that allow the operator to choose whether to take control or delegate it to the system (Yeo and Lin, 2020). Indeed the SoA has been suggested to objectively evaluate the quality of human-in-the-loop control for assistive technologies (Endo et al., 2020).

**Scenario example:** The user is translating a document from French to English using an autocompletion predictor (see Figure 9G). The user starts typing the first sentence and the system (which works with an extensive database) immediately predicts the most appropriate sentence in English, which is different than the sentence the user had in mind. The system shows the suggested sentence to the user who then approves it. The user continues translating the whole document and then starts losing the feeling of being assisted translated this document”). and thinks the decisions are fully made by themselves. When they finish, they attribute the outcome to themselves (e.g., “I translated this document”).

### Outcome augmentation (modulating the environment)

The SoA has been explained by retrospective theories suggesting that the experience of agency arises from variable *post-hoc* inferences occurring not only during the action but also after the action has occurred, rather than as a result of motor preparation and cognitive anticipation (Wegner, 2003). This means that the nature of the outcome can modulate the beliefs of the action (Johansson et al., 2005). Recent methods in HCI use outcome modulation, to create the illusion that an action’s outcome happening in the environment, is changed, or amplified. It is important to highlight that this technology does not change the physical environment itself but changes the beliefs about the environment when this cannot be physically changed. Although those illusions are usually unnoticed and they aim to match the user’s expectations, there is a causation conflict (what I did vs what it actually occurred) representing a shared agency between the user and the system, we call this *outcome augmentation*.

*Outcome augmentation* technology produces outcomes occurring exclusively outside the body (*external agency*). That is, systems modulate the experienced environment to match the expected outcome, aiming to give the user the perception amplified sensory features. We have mainly based our definition of *outcome augmentation* on changes in the environment in light of our literature review. We noted that HCI researchers have put considerable efforts on altering or influencing what the user experiences in virtual and real worlds. Algorithms change “reality” to give the user the perception of amplified sensory features (making a room bigger, an object heavier, one’s walking speed faster, one’s body thinner). While those features can relate to the body itself, we argue that the environment where the body is experienced is what is altered rather than the actual body itself. Therefore, we argue that *body* and *action augmentation* technology influences the user’s actions directly (e.g., making the user faster), while *outcome augmentation* technology influences the perception of the experienced environment influencing the user’s actions indirectly. For example, making the user believe the room is bigger even though the room dimensions remain unchanged in reality (Montano-Murillo et al., 2017), or making believe the user is walking faster (Interrante et al., 2007) while keeping a constant speed in reality.

Therefore, unlike *body augmentation*, where technology addresses limitations of the body itself, or a*ction augmentation* in which technology addresses limitations of the user’s skills, *outcome augmentation* addresses limitations of the experienced environment where the outcomes occur.

Providing the user with the expected outcome is straightforward when the physical environment allows to change it (e.g., illuminating the room when pressing the light switch). However, there are situations in which the physical environment cannot be changed or when the expected outcome is not possible to occur in the physical environment (e.g., touching objects that are not there, walking beyond the limits of a small room, changing objects textures). This is when *outcome augmentation* technology comes into play by modulating the beliefs about the outcome in the environment. That is, the user plans an action and expects an intended outcome, but since the environment cannot offer such outcome (e.g., due to constraints in the physical space) the system detects the environment limitations and adjusts it in a way that the user experiences a match between the action and the intended outcome.

**FIGURE 11:**
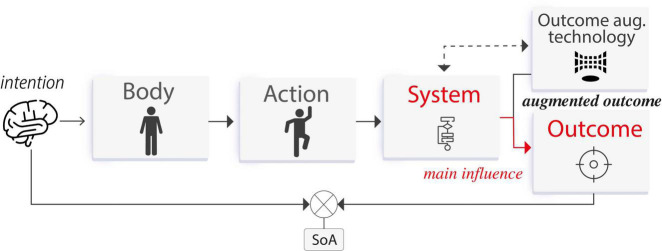
Agency process for *outcome augmentation* technology. The system modulates the outcome to match the user’s intention.

In this case, the SoA arises by a process in which the system modulates the outcome after the user executes an action. This outcome modulation is unnoticed by the user and therefore, they have an experience of agency. That is, the user observes the augmented outcome (conflicting the action) but still attributes it to their action. The main interaction is given by the influence that the system has over the outcome (see Figure 11).

**FIGURE 12:**
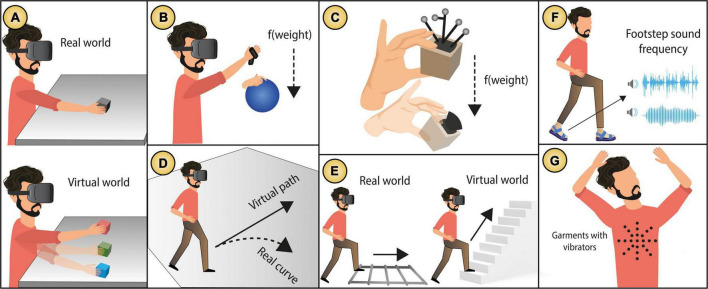
Examples of *outcome augmentation* technology: Retargeting techniques modulating **(A)** object quantity (Azmandian et al., 2016), **(B,C)** object weight (Rietzler et al., 2018; Samad et al., 2019), **(D)** navigation direction (Sharif et al., 2001), **(E)** navigation elevation (Nagao et al., 2018), and body perception **(F,G)** (Tajadura-Jiménez et al., 2019, 2020). Images from the original publications, fully attribution is given to the original authors.

**FIGURE 13:**
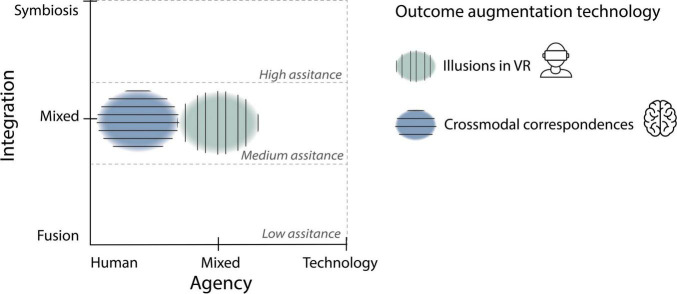
Map of the two types of *outcome augmentation* technology that we identified (Illusions in VR and crossmodal correspondences) and their relationship with assistance level, agency, and integration.

We identified two main types of *outcome augmentation* technology, illustrated in Figure 12, (1) *Illusions in VR*: integrated techniques in VR that by means of visual dominance, create the perception of amplified outcomes in the environment and (2) *Crossmodal correspondences*: integrated techniques that do not use VR but use cross-sensory associations to create the perception of amplified sensory features. See Figure 13 for a map of the two types of *outcome augmentation* technology that we identified and their relationship with assistance level (low-high), agency delegation (human-technology) and integration type (fusion-symbiosis).

#### Illusions in virtual reality

Integrated technology use VR to create illusions which are unnoticed by the user often taking advantage of the dominance of vision over touch (Rock and Victor, 1964) and motor cues (Salomon et al., 2016). Techniques in VR can be detached to the user’s body as many of them use mid-air interactions sensed by optical cameras (e.g., Kinect). Yet, many techniques use attached actuators integrated to the user’s body such as motion capture suits (Banakou and Slater, 2014) and headsets with attachments to the user’s face (Brooks et al., 2020).

Since this technology modulates beliefs about the outcome in the environment, it might also change the course of the user’s actions (e.g., modifying the user’s movements) by providing assistance in order to meet the intention. Therefore, this type of integration can be considered a mix of *fusion* and *symbiosis* as shown in Figure 13. That is, the user receives assistance from the system (mediated by body sensing) to execute actions, but the user always has an intention and acts in conjunction with the system (mixed agency).

Some examples of this technology are retargeting techniques that amplify limited conditions in the real environment. For example, Azmandian et al. (2016) used visual distortions to match the user intention of touching multiple objects while in reality only one was used (see Figure 12A). Rietzler et al. (2018) used visual redirections to make subjects believe an object was heavier that it actually was, thus meeting the user expectations of object weight (see Figure 12B). Similarly, Samad et al. (2019) manipulated the rendered position of the user’s hands—increasing or decreasing their displayed movements to induce weight perception without kinaesthetic feedback (see Figure 12C). Cheng et al. (2017) used passive haptics and hand redirecting to create the illusion of touching controllers and then meeting the user expectations of interacting with a cockpit. Zhao and Follmer (2018) explored haptic retargeting to minimise user-perceived difference between the physical proxy and virtual shape.

Using translational gains, these techniques can be even extended to modulate outcomes involving more complex actions such as walking, also called redirected walking (Razzaque et al., 2005). This technique is useful when the physical available space to walk is limited, and therefore helps meeting the user’s expectations during navigations tasks by modulating walking speed (Montano-Murillo et al., 2017), distance travelled (Sun et al., 2018) and walking elevation (Nagao et al., 2017). For example, Sun et al. (2018) proposed the “infinite walking” technique which gives the user the perception of walking larger distances than the available physical space (see Figure 12D). Similarly, Nagao et al. (2017, 2018) introduced the “infinite stairs” technique, giving the illusion of walking upstairs while actually walking on a flat surface (see Figure 12E). Some integrated systems combine redirection and motor actuation. For example, the “around the (Virtual) World” system (Auda et al., 2019), induces the effect of infinite walking using electric stimulation by actuating the legs of the user (stimulating the sartorius muscle) allowing the user to infinitely walk in the virtual world without the necessity to have an infinite physical world.

While the SoA is strictly factual (reflecting the experience of intention, movement, and outcome events as they occur), VR can produce agency beliefs that may be counterfactual. In other words, what the user does and what really happens may differ. Yet, multisensory stimulation provided by VR creates so realistic experiences that might produce strong outcome attribution (e.g., “I did that”) easily. Studies have shown that VR can produce outcome attribution even in absence of key elements that shape the SoA such as prior intention, feed-forward prediction, priming, and cause preceding effect (Banakou and Slater, 2014). This technology produces a strong sense of presence (Sanchez-Vives and Slater, 2005) so that, although the events are not real, people still experience both psychological and physiological responses to the events happening in the virtual environment. Taking advantage of this effect, *outcome augmentation* technology aims to modulate the beliefs about the outcome, matching the user’s intention, without diminishing the SoA when the environment conditions are limited.

**Scenario example:** The user is in a virtual world where they see three cubes in three different locations (Figure 12A) and then is asked to grab one by one. While grabbing each cube, they see how a virtual hand, matching their own hand’s position, travels different trajectories as they grab the different cubes. However, in the real world there is only one cube (Figure 12A). To create this illusion, an algorithm modifies the virtual seen trajectories to make the user believe there are three physical cubes in the real word. Although, in reality, the user moves her hand along the same trajectory for each cube, they do not notice such changes in her hand’s trajectories and therefore they experience a sensory match between her action and the seen resulting outcome and therefore a SoA occurs.

#### Crossmodal correspondences

Crossmodal correspondences (CCs) can provide perception of modified outcomes in the environment without the need of being immersed in a virtual world. CCs are defined “*as a tendency for a sensory feature, or attribute, in one modality, can be matched (or associated) with a sensory feature in another sensory modality*” (Spence and Parise, 2012). This associations have been widely employed in design and marketing. For instance, Van Doorn et al. (2017) found that the shape of a mug can influence the coffee taste expectations. Velasco et al. (2013) showed that the pouring sound can determine the temperature of a drink, and Reinoso Carvalho et al. (2016) suggest that certain music can modulate the taste attributes of beer. These crossmodal associations can serve to either augment or replace sensory features (Spence, 2018). For example, blind people might be able to listen to colours (Hamilton-Fletcher et al., 2016) or deaf people can feel music (Petry et al., 2018). Taking advantage of this crossmodal effect, CCs can be used to provide experiences of amplified sensory features when a certain sensory modality is limited.

While CCs have been studied extensively in psychology, their application to human–computer integration is less preeminent. However, we see an opportunity for technology designers to adopt CCs to provide the user with augmented sensory experiences and therefore meet the user’s expectations of an outcome. We already see some efforts in the literature. For example, Tajadura-Jiménez et al. (2019) combined ubiquitous wearable devices and sensory stimulation showing that altered footstep sounds can be used to change body perceptions during exertion exercise (e.g., lead people perceiving themselves as thinner/lighter, happier and walking more dynamically).

Another type of integrated technology is e-textiles which enables a *fusion* with the user’s body. In this line, Nava and Tajadura-Jiménez (2020) explored associations between haptic sensations produced by vibration patterns within textiles and “material perception” (e.g., rocks). They propose this “material perception” as a way to elicit different body perceptions (e.g., being heavy, strong). Similar effects were reported previously such as induced feeling of being “robotised” using vibration and sound accompanying the flexing of joints (Kurihara et al., 2013).

This type of integration can be considered a *fusion* as there is an embodied mediation in which sensors are attached to the user’s body (e.g., shoes that detect your walking patterns and change the sound of your footsteps). However, although the system does not act on behalf of the user (as intelligent systems do), it can influence the way people perceive their own body resulting in actual behavioural changes (making you walk faster or straighter). Therefore, we argue that this integration technology could be considered a mix of *fusion* and *symbiosis* in which agency is fully delegated to the user as there is low or null assistance provided by the system (see Figure 13).

While CCs are not too preeminent in the design of integrated technology, we included it in our classification because we see previous insights on how to use them for attached devices. Therefore, we aim to highlight the advantages of using CCs as the effect they produce looks promising for *outcome augmentation*. For example, we argue that for future integration technology using CCs, systems can modulate beliefs about the outcome in the environment, but not necessarily changing the course of the user’s actions (e.g., modifying the user’s movements) but influencing the feelings toward an outcome (e.g., feeling lighter, stronger, faster) in order to meet the intention. More opportunities around adopting CCs within human–computer integration technology can be studied in the future.

**Scenario example:** Imagine you want to improve your running time while training in the mornings, then a wearable smart t-shirt (through close-loop multisensory stimulation), makes you feel faster and stronger while running, consequently improving your actual performance. This improvement perception thus helps you meet your expectations of exercise completion at the same time it promotes a positive feeling toward exercising.

## Discussion and conclusions

In this paper, we have introduced a classification of the key elements that compose the SoA (*body*, *action* and *outcome*) and that technology designers aim to augment to give the user amplified experiences. We not only describe how agency arises in each of those categories, but also discern the type of agency experienced (body, external) as well as different examples of technologies fitting each category and their relation to integration (fusion, symbiosis) and agency delegation (human, technology).

We argue that the integration technology described in the different categories should always augment the user capabilities (e.g., improving speed, dexterity, productivity, etc.). That is, while a system could be seen as simply restoring a lost ability (e.g., a prosthesis that restore an amputation), the current physical attributes of the user should be augmented compared with their current constrains.

Moreover, while categories could overlap (as the exemplified in Figure 7), we mainly focussed on the markers of distinction of the different categories rather than in their similarities or overlaps. For example, a robotic arm could be augmenting the user’s body, their actions executed and perhaps the resulting outcomes as well. Therefore, we realised that we needed markers that delimit augmentation for integration technology. Based on the literature, we found that our categories have a (1) different role of agency (how SoA arises during the interaction), (2) different types of limitations that technology addresses (body, skills, environment) and (3) different agency type (body, external), as illustrated in Table 1. We consider these markers of differentiation more valuable to first, help partitioners identify their work within the integration research agenda, and second, to better define integration from the lens of agency.

In the next sections we describe advantages that represent opportunities for the future of integrated systems, as well as possible disadvantages representing ethical implications resulting from of the *symbiosis* between humans and computers. Bringing all this together, we conclude with an expanded definition of human–computer integration from the lens of agency.

### Opportunities for agentic integration

Integrated technology gives us the possibility to augment our own body, improve the performance of our actions and modulate our beliefs about the resulting outcomes. This effect can have many benefits for the user, not only when a sensory modality is reduced or limited but also when we simply want to improve our performance or when reality cannot offer a desired experience.

For example, *body* and *action augmentation*, in which technology is fused with the user’s body, benefits the area of “super humans” (augmenting people’s abilities) as well as the area of disabled humans (restoring missing functions). Imagine a rescuer using an exoskeleton to remove building debris and search missing people after an earthquake or for rover rescue missions on Mars (Palacios et al., 2021). This technology can also help to restore a missing or reduced SoA. For example, assisting in rehabilitation of motor functionality (Beckerle et al., 2017) or in conditions such as the alien hand syndrome (Badesa et al., 2014).

Moreover, in the case where technology is symbiosed with the user, considering agency implications for integrated technology can promote the design of responsible technology in the future. We live in a world in which integrated technology is becoming ubiquitous and increasingly digital, where researchers and engineers work on the digitalisation of the human senses (Velasco and Obrist, 2020) and the creation of the metaverse (Mystakidis, 2022). *Outcome augmentation* technology can help to meet the uses expectative in a digital world, in which the physical limitations constrain the user intentions. For example, making the user travel (Ranasinghe et al., 2018), or walk faster (Montano-Murillo et al., 2017), or to change their own body (Normand et al., 2011; Kilteni et al., 2012; Banakou et al., 2013, 2018; Serino et al., 2016). It is crucial however to highlight that the increasing usage of integrated technology also requires to consider responsibility in social contexts which raises some ethical concerns that we discuss in the next section.

### Ethical challenges of integration

Integrating humans with computers raises a number of ethical concerns. There could be situations in which the actions of an augmented user can be questioned. For instance, research has explored the idea to integrate the human body with technology in “superhuman sports” (Kunze et al., 2017). This might raise concerns of fairness of games when compared with non-augmented bodies. This can force sporting institutions to regulate the use of technology. For example, in the case of Paralympics and the use of bionic prosthetics (Richard et al., 2021). Another example is the use of “super humans” for military use, which has raised legal concerns (Shah, 2019).

In light of the improved performance that technology can bring, integrated systems could also be addictive. The more technology is integrated to our body and daily lives, the more we are likely to become addicted to some kind of device due to an increase in our productivity (Turel et al., 2011; Washington, 2021). The responsible innovation framework by Stilgoe et al. (2020) tells us that we need to anticipate potential problems that come with any innovation. Therefore, technology designers have to explore what new problems may manifest in society before introducing new technology. For example, the introduction of autonomous cars required new road and legal regulations (Beiker, 2012; Harel et al., 2020), while the introduction of social media saw an increase in cyberbullying (Whittaker and Kowalski, 2015) which in turn forced institutions to regulate digital content (Piccoli et al., 2020). Similarly, the introduction of new integrated systems could produce implicit and unplanned issues that need to be anticipated in order to avoid them or create appropriated regulations and therefore promote responsible innovations.

Moreover, responsibility becomes crucial in autonomous systems. Assistance levels given to the user need to be carefully designed since increased automation can lead to the question—who is in control now? (Berberian et al., 2012). While causality and accidents are usually attributed to human errors, today crucial actions (e.g., driving in public roads) have been delegated to computers. Therefore, it is important that automated systems give users the appropriate feeling of control in order to preserve the feeling of control.

Another ethical consideration is around body data usage. Usually, technology companies ask their users to consent sharing their information such as name, address, affiliation, etc. (Zimmer, 2020). Integrated technology, being so close to the user body (involving biosensing), often uses data recordings of biological functions. This data recording could need regulation in the future. For example, would you give your consent to share your brain activity with a technology company?

### A new perspective from the lens of agency

The concept of integration has been introduced many years ago, using terms such as “cooperation” or “partnership” between humans and computers (Licklider, 1960; Clark, 2001; Engelbart, 2001). The most recent articulations have been proposed by Farooq and Grudin (2016)—a *symbiosis* which occurs when agency is shared between humans and digital systems as they assist or work on the humans’ behalf, and by Mueller et al. (2020)—a sensory *fusion* between the user and computers, in which the system understands the user’s implicit precognitive needs through bio-sensing, and communicates directly to human senses. We expand upon those recent views to include other aspects related to agency, which we consider to be particularly relevant. For example, in the presented classification of different integrated technologies, we suggest that both views are valid, but we further argue that the type of integration (fusion, mixed, symbiosis) varies depending on the level of agency experienced (human—mixed—technology).

For designers introducing novel integrated technologies, it could be confusing to identify which term is more suitable to use. Wearable systems can be *fused* with the user’s body, but they can also act on the human’s behalf representing a *symbiosis*. We suggest that a boundary that divides them, is the level of agency experienced. For example, for integrated technology where there is a *fusion*, agency is usually higher, which means that the user has more degree of control over the system, as is the case of *body augmentation* technology (extra limbs and prosthetics). For integrated technology representing more a *symbiosis*, agency is usually lower, which means that the system assists and often acts on behalf the user, as is the case of *action augmentation* technology (e.g., motor actuation and autonomous systems).

We also identify that the type of agency (*body* and *external*) influences integrated technology. For example, for *body augmentation* technology, the agency experienced is internal, with outcomes being body movements or processes involving the body itself (e.g., proprioception). For *action augmentation* technology, both *body* and *external* agency can be experienced. That is, systems can provide outcomes inside or outside the body. For *outcome augmentation* technology, systems mainly produce *external* agency, which means that outcomes occur in the external environment.

Therefore, in light of the present review, and building upon the recent views from Farooq and Grudin (2016) and Mueller et al. (2020), we argue that human–computer integration is a partnership between humans and technology, in which systems augment the capabilities of the user’s *bod*y, their *actions*, and the resulting *outcomes*. In this partnership, a SoA is shared through a sensory *fusion*, but also through a *symbiosis*. However, the more devices are fused to the user’s body (*fusion*) the more control humans have over the system. Similarly, the more the integrated technology acts on the human’s behalf (*symbiosis*) the less control the human has over the system. A *fusion*-*symbiosis* trade-off that HCI researchers and practitioners need to balance. Additionally, we suggest designers and researchers to think about the type of limitation that a novel integrated system aims to solve. Identifying where the limitation comes from (user’s body, user’s skills, or the experienced environment) can help to identify the type of augmentation required (*body*, *action*, *outcome*) which in turn can help to identify the type of agency that will be experienced (*body* or *internal*).

We hope these considerations and markers of differentiation involving SoA within current integrated systems can help researchers, designers, and practitioners to better situate their work and consider a feeling of being in control for future integrated technology.

## Author contributions

PC: methodology, conducting the review, interpretation, and writing—original draft preparation and review and editing. PH: interpretation and writing—review and editing. KH: interpretation and writing—review and editing. OG, JB, and SS: writing—review and editing. MO: supervision, interpretation, and writing—review and editing. All authors contributed to the article and approved the submitted version.
